# Rapid reconstruction of multidrug‐resistant bacterial genomes using CycloneSEQ nanopore sequencing

**DOI:** 10.1002/imo2.70060

**Published:** 2025-10-26

**Authors:** Kai Peng, Zeyu Tong, Changan Li, Zhichao Li, Xin Lu, Cemil Kurekci, Mashkoor Mohsin, Zhiqiang Wang, Yong‐Xin Liu, Ruichao Li

**Affiliations:** ^1^ Jiangsu Co‐Innovation Center for Prevention and Control of Important Animal Infectious Diseases and Zoonoses, College of Veterinary Medicine Yangzhou University Yangzhou China; ^2^ Institute of Comparative Medicine Yangzhou University Yangzhou China; ^3^ National Institute for Communicable Disease Control and Prevention Chinese Center for Disease Control and Prevention Beijing China; ^4^ Department of Food Hygiene and Technology, Faculty of Veterinary Medicine Hatay Mustafa Kemal University Hatay Türkiye; ^5^ Department of Microbiology, Faculty of Veterinary Medicine Dokuz Eylül University Izmir Türkiye; ^6^ Institute of Microbiology University of Agriculture Faisalabad Faisalabad Pakistan; ^7^ Genome Analysis Laboratory of the Ministry of Agriculture and Rural Affairs, Agricultural Genomics Institute at Shenzhen Chinese Academy of Agricultural Sciences Shenzhen China

## Abstract

Multidrug‐resistant (MDR) bacteria commonly harbor highly complex genomes enriched with mobile elements and resistance islands, which are challenging to resolve using short‐read sequencing. Here, we evaluated CycloneSEQ, a newly developed Chinese nanopore sequencing platform, for MDR genome reconstruction. Long‐read‐only assemblies generated near‐complete genomes, while hybrid assemblies achieved accuracy exceeding 99.99%. CycloneSEQ effectively resolved complex structures, including tandem repeats within ARG‐bearing regions. Notably, updated sequencing chemistry improved single‐read accuracy to 96.2% and yielded assembled genomes with 99.98% accuracy. These results demonstrate that CycloneSEQ enables the generation of complete and highly accurate bacterial genomes, highlighting its potential for antimicrobial resistance research and clinical surveillance.

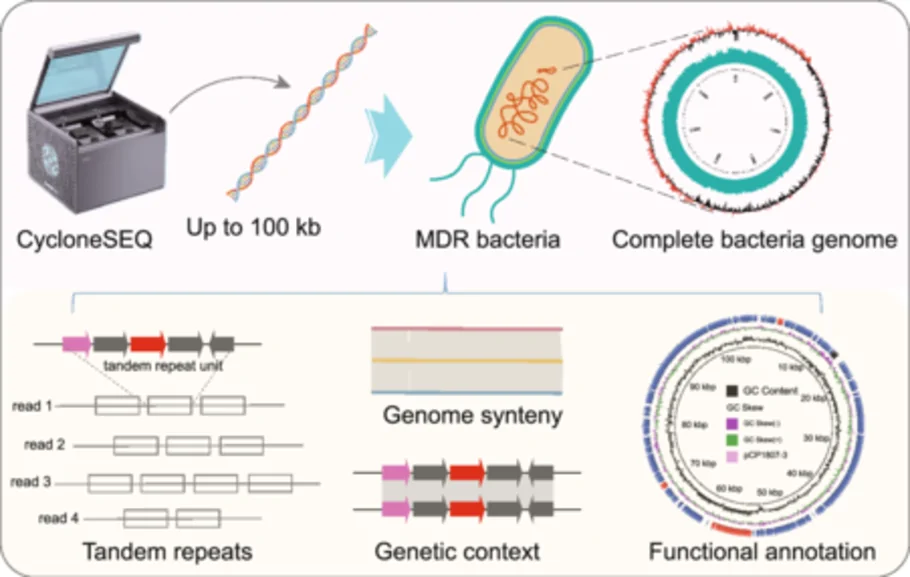

To the Editor,

Bacteria serve fundamental roles in maintaining ecosystem balance and supporting human health [1, 2]. However, pathogenic strains can cause severe infections in humans and animals, further complicated by their rapid evolution of antibiotic resistance (AMR) [1, 2, 3, 4]. This issue is particularly pronounced in multidrug‐resistant (MDR) bacteria, which often harbor highly complex genomes characterized by a high abundance of mobile genetic elements, resistance islands, and repetitive sequences [5, 6, 7]. These features make it difficult to resolve MDR bacterial genomes using conventional high‐throughput short‐read sequencing technologies, which struggle to span repetitive or structurally variable regions [8]. Hence, long‐read sequencing technologies, including Oxford Nanopore Technology (ONT) nanopore sequencing and PacBio Single Molecule Real‐Time sequencing have been widely used to unravel the genetic structures of MDR bacteria [9, 10, 11]. Recently, MGItech, a leading Chinese genomics company, launched its first nanopore sequencing platform, CycloneSEQ. However, the performance of CycloneSEQ in resolving the genetic structures of MDR bacteria remains unknown. In this study, we employed the CycloneSEQ nanopore sequencing platform to resequence multiple MDR bacterial species, evaluated the impact of different bioinformatics methods on genome reconstruction, and provided a comprehensive assessment of its potential utility in antimicrobial resistance research and clinical applications.

MDR plasmids, integrative and conjugative elements (ICEs), integrons, and transposons are common genetic elements that facilitate the horizontal transfer of ARGs [12]. MDR bacterial strains often harbor multiple such elements, contributing to highly complex genomic structures that pose significant challenges for accurate genome assembly and analysis [13]. To evaluate the performance of CycloneSEQ for MDR bacterial genome reconstruction, we selected 10 MDR strains, each harboring multiple ARGs‐bearing mobile elements, and performed resequencing and analysis. Among them, the structures of several representative MDR plasmids are presented in Figure S1. Moreover, some genomes harbored complex genetic structures, including tandem repeat sequences. The detailed genetic characteristics of all tested strains are provided in Table S1.

## 1 THE FEATURE OF CYCLONESEQ SEQUENCING DATA

We obtained a total of 9.35 Gb of nanopore sequencing data with a *Q* value greater than 7 from all sequenced strains. Read length distribution analysis revealed that a large proportion of reads had lengths ranging from 10 to 20 kb, and a subset of exceptionally long reads (>100 kb) was also detected (Figure S2A,B). We observed a typical inverse correlation between read length and total sequencing throughput, with no evidence of read count enrichment at any specific read length (Figure S2B). Subsequently, we analyzed the relationship between read length and average base quality. The results showed that longer sequencing reads generally exhibited higher base quality (Figure 1A). Most reads longer than 10 kb had a *Q* value higher than 10 (Figure 1A). Using barcode‐specific demultiplexing, we obtained genome‐specific sequencing data ranging from 736 to 1124 Mb (with sequencing read numbers ranging from 186,259 to 402,930) per bacterial strain (Figure S2C,D). Read length N50 varied across samples, with ZN2 showing the highest N50 (12 kb) and IC7‐2 the lowest (7 kb) (Figure 1B). Notably, read length distributions and average base quality were consistent across all samples (Figure S2E,F).

**FIGURE 1:**
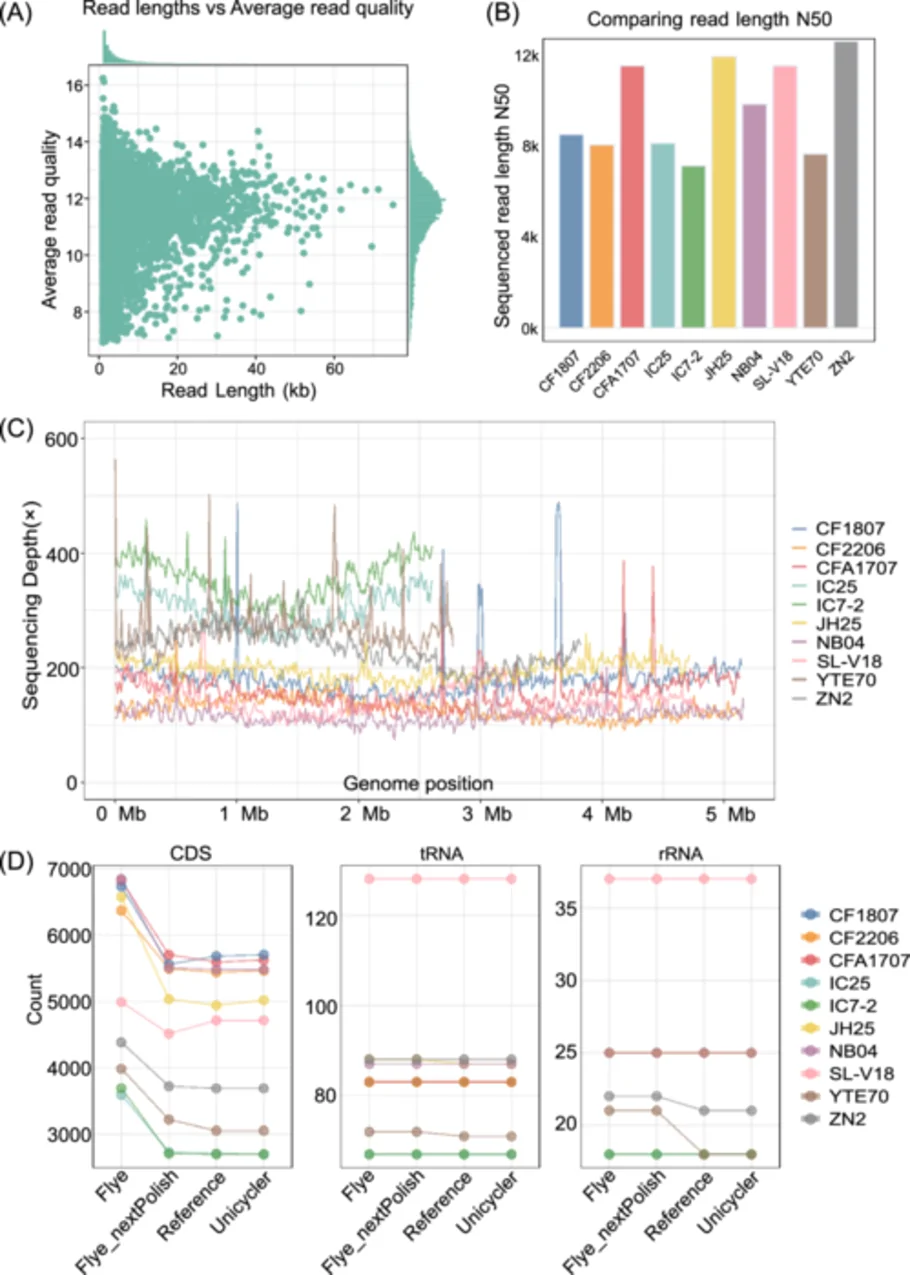
Sequence features of CycloneSEQ nanopore sequencing data and the assembly quality of bacterial genomes using CycloneSEQ nanopore sequencing. (A) Comparison of sequence length and sequence Phred quality score of CycloneSEQ nanopore sequencing data. (B) Comparison of the read N50 of each sample. (C) The sequence homogeneity of CycloneSEQ nanopore sequencing for different samples. (D) Comparison of the predicted count of CDS, tRNA, and rRNA in the bacterial genomes obtained by long‐read assembly, polishing, and hybrid assembly with those in the reference genomes.

## 2 THE ALIGNMENT HOMOGENEITY AND SEQUENCING READ ACCURACY

In contrast to conventional short‐read sequencing, which typically involves PCR amplification during library preparation, nanopore sequencing, including CycloneSEQ, can be performed without such an additional step. Therefore, sequencing bias typically observed in short‐read sequencing does not occur in CycloneSEQ nanopore sequencing. To verify this, we mapped the sequencing data to reference complete genomes (constructed by our previous studies). Notably, plasmid sequences, which are extrachromosomal DNA elements with typically uncertain copy numbers, were excluded from the analysis because their sequencing depth is often inconsistent with that of the chromosomal genome. By analyzing the mapping results, we found that there were no sequencing gaps in any of the genomes (Figure 1C). Additionally, the sequencing depth for each bacterial genome was generally consistent, and no sequencing bias was detected (Figure 1C). We then performed single‐read accuracy analysis on the filtered data using an alignment strategy. The raw sequencing data of each bacterial genome were mapped to their respective reference genomes using minimap2. The average read accuracy was calculated to be over 92%, with sample ZN2 having the highest accuracy at 93.19%, and sample CFA1707 with the lowest at 92.06% (Figure S3). Overall, the accuracy of CycloneSEQ nanopore sequencing is comparable to that of previous ONT and QitanTech nanopore sequencing platforms.

## 3 BACTERIAL GENOME ASSEMBLY AND POLISHING WITH DIFFERENT STRATEGIES

After evaluating the quality of the raw nanopore sequencing data, bacterial genomes were assembled using long‐read assembly tools including Canu, Flye, NextDenovo, and wtdbg2. We found that the majority of bacterial genomes were assembled into a few contigs using Flye, NextDenovo, and wtdbg2, which were very close to complete bacterial genomes (Figure S4). Whereas Canu usually produces a fragmented genome (Figure S4). Although NextDenovo and wtdbg2 were not originally designed for bacterial genome assembly, both performed well in assembling bacterial chromosomes but showed reduced effectiveness in plasmid assembly. Many plasmids, especially for plasmids with lengths shorter than 50 kb, were lost after de novo assembly (Table S2). Among all assemblies, sample YTE70 produced the poorest result, as its chromosome could not be assembled into a complete circular form. Further detailed analysis revealed the presence of a chromosomally integrated plasmid of approximately 33 kb within the chromosome assembly (Figure S5). Notably, while the genomes assembled using only long‐read sequencing were continuous, the Average Nucleotide Identity showed a significant difference compared to the reference genomes. Therefore, high‐accuracy short‐read sequencing is typically used for polishing long‐read assembled genomes. Pilon and NextPolish, two commonly used polishing tools, were applied here for genome polishing. After polishing, the ANI of all long‐read assembled genomes exceeded 99.99% when compared with the reference genomes (Figure S6A). Our analysis further confirmed that both Pilon and NextPolish significantly improved the accuracy of the long‐read assembled genomes.

Currently, mainstream hybrid assemblers generally assemble short‐read data first and then optimize the assembly graph using long‐read data [14]. Therefore, long‐read data has a significant impact on the quality of hybrid assembled genomes. We used Unicycler to assemble the CycloneSEQ long‐read data and Illumina short‐read data. We found that the majority of genomes were assembled relatively continuous, except for samples NB04 and ZN2 (Table S2). This demonstrates that CycloneSEQ long‐read data can significantly improve the assembly graph generated from short‐read data assembly.

## 4 QUALITY ASSESSMENT OF ASSEMBLED GENOMES

To evaluate the quality of the assembled genomes, various quality assessment indicators were tested. First, we compared the genome sizes and GC content of the assembled genomes with those of the reference genomes. The result showed that most size differences were within 50 kb, and GC content deviations were within 0.02% (Figure S6A). In contrast, the assembly of strain NB04 exhibited a >100 kb discrepancy, which was traced to the loss of a 140 kb chromosomal segment during hybrid assembly. The genome size and GC content of the bacterial genomes generated by hybrid assembly were closer to the reference genomes. Next, we analyzed the number of annotated CDS, tRNA, and rRNA in the long‐read assembled genomes, long‐read assembled error‐corrected genomes, and hybrid assembled genomes. The results showed that long‐read‐only assembled genomes resulted in many incorrect predictions for CDS, tRNA, and rRNA (Figure 1D). In comparison, polished genomes exhibited more accurate predictions for these elements (Figure 1D). Finally, genome synteny was analyzed. The majority of assembled genomes showed strong synteny with the reference genome, maintaining conserved gene order and orientation (Figure S6B).

## 5 THE INFLUENCE OF SEQUENCE DEPTH ON THE GENOME ASSEMBLY AND THE ACCURACY OF THE ASSEMBLED GENOME

The sequencing depth of bacterial genomes can significantly affect the completeness and accuracy of the assembled genomes. Strain IC7‐2, which had the highest sequencing depth, was chosen for this analysis. We then randomly subsampled its sequencing data to different depths and performed separate assemblies. The results demonstrated that a relatively complete bacterial genome, with an ANI of 98.88%, could be obtained using only nanopore long‐read sequencing data at a depth of 15× (Figure S7). As the sequencing depth increased, the accuracy of the assembled genome improved. However, beyond a depth of 50×, the accuracy of the assembled genomes did not show further improvement (Figure S7). When using a hybrid assembly strategy to assemble the IC7‐2 genome, a complete genome could be obtained with a long‐read sequencing depth of 5× (Figure S7). This demonstrates that CycloneSEQ nanopore sequencing data significantly enhances genome assembly quality when using hybrid assembly strategy. More importantly, the genome generated through hybrid assembly showed 100% identity with the reference. Additionally, we found that the quality of the hybrid assembly genome remained unchanged as the long‐read sequencing depth increased.

## 6 THE ABILITY TO RESOLVE COMPLEX GENETIC STRUCTURES

The genomes of MDR bacteria often contain complex structures that challenge conventional long‐read sequencing. In particular, heterogeneous tandem repeats hinder accurate assembly and are closely linked to resistance phenotypes, as changes in repeat copy number can alter ARG expression and the stability of mobile elements. For example, the tandem repeat heterogeneity of *poxtA* in strain IC25 remains difficult to resolve. To verify the capability of CycloneSEQ in analyzing the genetic heterogeneity of tandem repeat structures, we conducted a detailed single‐read analysis on the raw nanopore sequencing data of strain IC25. The results demonstrated that many sequencing reads could span the *poxtA*‐bearing repeat regions (Figure 2A). Further analysis showed that most of the reads spanned only one or two *poxtA*‐carrying repeat units. The length of these reads was basically less than 50 kb (Figure 2B). In addition, many reads with three or more tandem repeat units were found, and these reads were longer. Subsequently, we analyzed the Phred quality score of all reads carrying repetitive units of the *poxtA* gene. The results showed that the majority of reads had Phred quality score between 9 and 11 (Figure 2B). Notably, when we analyzed some of ultra‐long reads, we found some more complex tandem repeat structures. Normally, one tandem repeat unit consist of two IS*1216E*, two *poxtA* and one *fexB*. We found that some of raw reads only carried the IS*1216E*‐*poxtA*‐IS*1216E* structure, which was not complete tandem repeat unit. In addition, we found that multiple tandem repeat units in a read were truncated by other genetic elements, which also appeared to be mediated by IS*1216E*.

**FIGURE 2:**
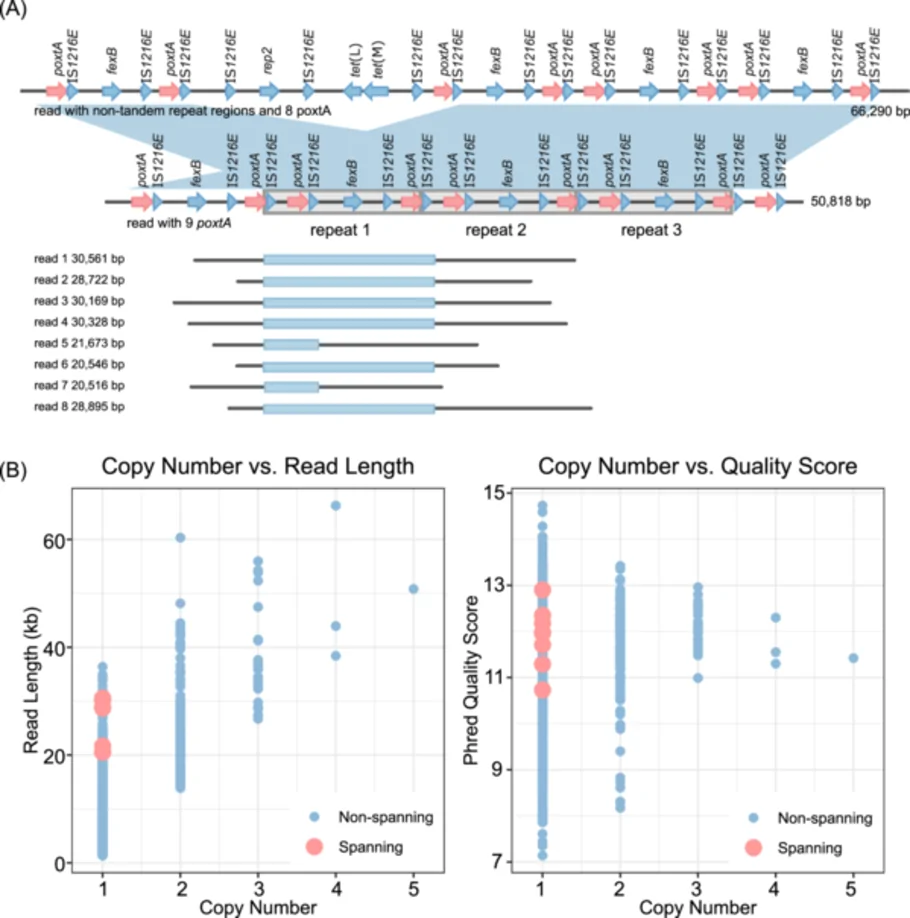
The ability of CycloneSEQ nanopore sequencing data in resolving the structure and heterogeneity of gene tandem repeats. (A) Tandem repeats of *poxtA* gene in strain IC25. (B) The read length and read quality distribution of *poxtA*‐bearing reads.

## 7 IMPROVEMENT IN THE ACCURACY OF CYCLONESEQ SEQUENCING

Recently, CycloneSEQ has improved its sequencing chemistry, leading to a substantial enhancement in sequencing accuracy. We evaluated the new chemistry using an MDR *E. coli* strain (yzc_w3_1) and found that the average read accuracy of the latest CycloneSEQ platform increased to 96.16%, with the raw Phred quality score improving to 17.1, while the read length distribution remained comparable to that of the previous CycloneSEQ chemistry (Figure S8). We further compared the accuracy of the genome assembled using only CycloneSEQ reads with that obtained through hybrid assemblies combining CycloneSEQ and MGI DNBSEQ short‐read data. The genome assembled exclusively with CycloneSEQ reads contained no misassemblies, only a few mismatches, and achieved an overall accuracy of 99.98% (Figure S9). These results indicate that CycloneSEQ has the potential to generate complete and highly accurate bacterial genomes without the need for short‐read polishing. Looking ahead, further improvements such as optimized library preparation chemistry, more advanced basecalling algorithms, and cost reduction strategies will likely enhance the platform's utility and accessibility, ultimately expanding its applications in microbial genomics and clinical microbiology.

In conclusion, our study provides a comprehensive evaluation of the CycloneSEQ nanopore sequencing platform for MDR bacterial genome assembly. We demonstrated that CycloneSEQ long‐read data enable the reconstruction of near‐complete bacterial genomes, resolve complex genetic structures, and improve the quality of hybrid assembly. These findings suggest that CycloneSEQ represents a promising tool for bacterial genomics, particularly in antimicrobial resistance research, where high‐resolution genome assembly is essential. In addition, future advancements in CycloneSEQ long‐read sequencing technology and bioinformatics tools will further enhance our ability to decipher the genetic landscapes of MDR bacteria [15].

## AUTHOR CONTRIBUTIONS

**Kai Peng**: Conceptualization; methodology; software; investigation; visualization; funding acquisition; writing—original draft; writing—review & editing. **Zeyu Tong**: Software; investigation; visualization. **Changan Li**: Investigation. **Zhichao Li**: Investigation. **Xin Lu**: Supervision. **Cemil Kurekci**: Writing—original draft. **Mashkoor Mohsin**: Writing—original draft. **Zhiqiang Wang**: Writing—review & editing; project administration; supervision; funding acquisition. **Yong‐Xin Liu**: Conceptualization; supervision; project administration; writing—review & editing. **Ruichao Li**: Writing—review & editing; project administration; supervision; funding acquisition. All authors have read the final manuscript and approved it for publication.

## CONFLICT OF INTEREST STATEMENT

The authors declare no conflicts of interest.

## ETHICS STATEMENT

No animals or humans were involved in this study.

## Supporting information


**Figure S1.** The genetic structures of the plasmids in strains CF1807, NB04, SL‐V18 and ZN2.
**Figure S2.** The features of CycloneSEQ nanopore sequencing reads.
**Figure S3.** The reads accuracy of different samples produced by CycloneSEQ.
**Figure S4.** The assembly quality of different samples with different assembly tools.
**Figure S5.** Comparative analysis of genome YTE70 assembled using different assembly tools.
**Figure S6.** Evaluation of assembly quality of different samples.
**Figure S7.** The influence of sequencing depth on the integrity and accuracy of bacterial genome assembly.
**Figure S8.** Key features of sequencing data produced by the updated CycloneSEQ sequencing chemistry.
**Figure S9.** Quality assessment of strain yzc_w3_1.

**Table S1.** The genome information of MDR bacteria in this study.
**Table S2.** Comparative analysis of assembled contigs by various software tools with the reference genomes.

## Data Availability

The raw CycloneSEQ sequencing data have been deposited in the NCBI SRA database under BioProject accession PRJNA1315208 (https://www.ncbi.nlm.nih.gov/bioproject/PRJNA1315208/). The data and scripts used in this study are saved in GitHub (https://github.com/P-kai/iMetaOmics-CycloneSEQ). Supplementary materials (methods, figures, tables, graphical abstract, slides, videos, Chinese translated version, and update materials) may be found in the online DOI or iMetaOmics http://www.imeta.science/imetaomics/.
