# Supplementary material for: Rapid reconstruction of multidrug‐resistant bacterial genomes using CycloneSEQ nanopore sequencing
Source: IMetaOmics. 2025 Oct 26;3(1):e70060. doi: 10.1002/imo2.70060 (PMC13674401; doi:10.1002/imo2.70060)
Supplement: Supplementary file 1 [file IMO2-3-e70060-s001.docx]

**Supporting information to Rapid reconstruction of multidrug resistant bacterial genomes using CycloneSEQ nanopore sequencing**

**Running title:** CycloneSEQ sequencing in bacterial genomes

Kai Peng^1,2^, Zeyu Tong^1,2^, Changan Li^1,2^, Zhichao Li^1,2^, Xin Lu^3^, Cemil Kurekci^4, 5^, Mashkoor Mohsin^6^, Zhiqiang Wang^1,2*^, Yong-Xin Liu^7*^, Ruichao Li^1,2*^

^1^Jiangsu Co-Innovation Center for Prevention and Control of Important Animal Infectious Diseases and Zoonoses, College of Veterinary Medicine, Yangzhou University, Yangzhou, China

^2^Institute of Comparative Medicine, Yangzhou University, Yangzhou, China

^3^National Institute for Communicable Disease Control and Prevention, Chinese Center for Disease Control and Prevention, Beijing, China

^4^Department of Food Hygiene and Technology, Faculty of Veterinary Medicine, Hatay Mustafa Kemal University, Hatay, Türkiye

^5^Department of Microbiology, Faculty of Veterinary Medicine, Dokuz Eylül University, Izmir, Türkiye

^6^Institute of Microbiology, University of Agriculture, Faisalabad, Pakistan

^7^Shenzhen Branch, Guangdong Laboratory of Lingnan Modern Agriculture, Genome Analysis Laboratory of the Ministry of Agriculture and Rural Affairs, Agricultural Genomics Institute at Shenzhen, Chinese Academy of Agricultural Sciences, Shenzhen, China

**^*^Correspondence**: Ruichao Li (rchl88@yzu.edu.cn); Yong-Xin Liu ([liuyongxin@caas.cn);](mailto:liuyongxin@caas.cn);) Zhiqiang Wang (zqwang@yzu.edu.cn)

**Materials and methods**

**Samples collection and DNA extraction**

A total of 10 strains with different species were collected from our laboratory and our partner laboratories. These strains are typical of clinically important MDR bacteria and carry many critical ARGs. In addition, the genomes of these strains are complex, making them ideal for evaluating the feasibility of long-read sequencing technology. The detail information of the 10 strains was presented in Table S1. Of note, all of these strains have been well investigated in our previous studies. After bacteria collection, all strains were recovered using different bacterial culture media. Then, cultured bacteria were subjected to DNA extraction using FastPure Bacteria DNA Isolation Mini Kit (Vazyme) following the standard protocol. Extracted bacterial genome DNA were stored at -80 ℃ for further study.

**Genomic DNA quality control**

Before DNA sequencing, the purity and concentration of extracted genome DNA were tested using NanoDrop 2000, gel electrophoresis and Qubit® Fluorometer. Briefly, we first used gel electrophoresis to confirm that the DNA fragments were larger than 15 kb. Then, we used NanoDrop 2000 to confirm a 260/280 ratio of ~1.8 for extracted DNA. At last, the concentration of genomic DNA was determined using a dsDNA Broad-Range Assay kit on the Qubit® Fluorometer. The total DNA production should be enough for both short-read and long-read nanopore sequencing.

**Short-read and long-read sequencing**

After quality control, all bacterial genomic DNA were subjected to short-read and long-read sequencing. Short-read sequencing was performed at BGI in Nanjing using the BGIseq-500 platform with a paired-end read strategy (2×150 bp). In parallel, long-read sequencing was performed on the latest MGI CycloneSEQ platform. Detailed technical parameters about MGI CycloneSEQ can be obtained at https://en.mgi-tech.com/Home/Applications/index/id/20.html.

For CycloneSEQ long-read library preparation, approximately 2 mL of bacterial culture (~10^8^-10^9^ cfu) was extracted using a high-throughput DNA extraction kit. Two μg of purified DNA were diluted and mixed with DNA repair buffers and enzymes, followed by incubation at 20 ℃ and 65 ℃ for 10 min each. The repaired DNA was purified using 1.0 × DNA clean beads and eluted in 240 µL nuclease-free water. For adaptor ligation, end-repaired DNA was combined with sequencing adaptors, ligation buffer, and ligase, then incubated at 25 ℃ for 30 minutes. Ligated products were again purified, washed with long fragment wash buffer, and eluted in 42 µL of elution buffer. The final libraries were quantified using a Qubit fluorometer and sequenced on the CycloneSEQ WuTong02 platform according to the manufacturer’s protocol. This streamlined workflow enabled efficient preparation of long-read sequencing libraries for bacterial genome assembly. Overall, the total time required for library construction is approximately 1.5 hours, and the sequencing run takes about 8 hours. Therefore, when combined with an established bioinformatics analysis pipeline, a complete bacterial genome can be obtained within as little as 12 hours.

**CycloneSEQ Long-Read Quality Evaluation**

We first assessed the quality of sequencing reads from all samples generated by the CycloneSEQ nanopore sequencer using NanoPack v1.25, a toolkit designed for evaluating long-read sequencing data [1]. Parameters such as read length N50, average base quality score, and mean read length were analyzed. The quality of the data across different samples was evaluated with Nanoplot, and a comparative analysis of the data quality between samples was conducted using NanoComp. The alignment characteristics of the original CycloneSEQ sequencing data were generated with minimap2 and LAST, and the results were summarized and visualized with Qualimap2 v2.2.1 [2-4].

**Genome Assembly and Polishing**

Complete bacterial genomes are typically generated by using short-read data to refine draft assemblies constructed from long reads or through a hybrid assembly approach that integrates both short-read and long-read data [5]. In this study, we employed both methods to evaluate the effectiveness of CycloneSEQ long-read data in assembling complete bacterial genomes. Short-read sequencing data were assembled using SPAdes with a coverage threshold of 30 [6]. For long-read assembly, we used Flye, Canu, nextDenove and wtdbg2, common available assemblers for long-read sequencing data [7-10]. Genome polishing was performed with Pilon and NextPolish, leveraging high-accuracy second-generation sequencing data [11, 12]. Additionally, a hybrid assembly combining CycloneSEQ long-read data and BGI short-read data was conducted using Unicycler [13]. To mitigate potential issues arising from low-quality long reads, CycloneSEQ long-read data were filtered with SeqKit v0.8.0 before proceeding with the hybrid assembly [14].

**Genome Integrity and Accuracy Assessment**

To evaluate the quality of assembly results, we first counted the reports generated by different assemblers and visualized them using Bandage v0.8.1 [15]. Genome integrity and accuracy were then assessed with QUAST, using a standard complete genome as a reference [16]. The accuracy of genomes assembled with CycloneSEQ long reads was further evaluated using dnadiff from the MuMmer toolkit [17]. Additionally, for the strain with the highest sequencing depth, we compared randomly sampled sequences from the CycloneSEQ raw data with the reassembled genome to assess the impact of sequencing depth on the final assembly quality.

**Genome Annotation and ARGs Identification**

The bacterial genomes assembled from CycloneSEQ sequences were functionally annotated using the RAST server (http://rast.nmpdr.org/), with manual refinements [18]. ARGs and insertion sequences were identified using abricate with ResFinder and PlasmidFinder databases [19, 20]. Plasmids were visualized and compared using BRIG and Easyfig [21, 22].

Figure S1. The genetic structures of the plasmids in strains CF1807, NB04, SL-V18 and ZN2. (A) An MDR plasmid co-harboring *bla*_KPC-2_ and *tmexC2D2-toprJ2* in strain CF1807. (B) A multi-replicon plasmid from strain NB04 carrying *tmexC2D2-toprJ2*, with replicon types IncHI1B and IncFIB(Mar). (C) A *bla*_VMB-2_-positive plasmid from strain SLV-18. (D) A *tet*(X6)-bearing IncA/C plasmid from strain ZN2.

Figure S2. The features of CycloneSEQ nanopore sequencing reads. (A, B) The distribution of read lengths. (C, D) Comparisons of the total number of sequencing reads and sequencing throughput of each sample. (E, F) Comparisons of the read length and quality of sequencing data obtained from different samples.

Figure S3. The reads accuracy of different samples produced by CycloneSEQ. Sample ZN2 has the highest accuracy at 93.19%, whereas sample CFA1707 has the lowest accuracy at 92.06%

Figure S4. The assembly quality of different samples with different assembly tools. The comparisons of genome size, GC content, contig N50, and other assembly metrics were generated using QUAST.

Figure S5. Comparative analysis of genome YTE70 assembled using different assembly tools. The orange-colored region indicates the plasmid sequence that was incorrectly assembled into the chromosome.

Figure S6. Evaluation of assembly quality of different samples. (A) A comparison between the reference genome and the bacterial genomes derived by hybrid assembly, long-read assembly, and polishing in terms of genome size, GC content and genome accuracy. The genome similarity was calculated using ANI. (B) Collinearity analysis of long-read assembled genomes and hybrid assembled genomes with reference genomes.

Figure S7. The influence of sequencing depth on the integrity and accuracy of bacterial genome assembly. As sequencing depth increased, the accuracy of the assembled genome improved. However, once the depth exceeded 30 ×, further increases had little effect on enhancing assembly accuracy.

Figure S8. Key features of sequencing data produced by the updated CycloneSEQ sequencing chemistry. (A) Comparison of sequence length and sequence quality values of CycloneSEQ nanopore sequencing data. (B) The distribution of read lengths.

Figure S9. Quality assessment of strain yzc_w3_1. (A) Comparison of genomic features between the Flye assembled genome and the reference genome. (B) Comparison of contigs length between the Flye assembled genome and the reference genome.

References:

1. Coster, Wouter De, Rosa Rademakers. 2023. “NanoPack2: population-scale evaluation of long-read sequencing data.” *Bioinformatics* 39:btad311. <https://doi.org/10.1093/bioinformatics/btad311>

2. Li, Heng. 2018. “Minimap2: pairwise alignment for nucleotide sequences.” *Bioinformatics* 34: 3094-3100. <https://doi.org/10.1093/bioinformatics/bty191>

3. Sahl, Jason W, J Gregory Caporaso, David A Rasko, Paul Keim. 2014. “The large-scale blast score ratio (LS-BSR) pipeline: a method to rapidly compare genetic content between bacterial genomes.” *PeerJ* 2:e332. https://doi.org/10.7717/peerj.332

4. Okonechnikov, Konstantin, Ana Conesa, Fernando García-Alcalde. 2016. “Qualimap 2: advanced multi-sample quality control for high-throughput sequencing data.” *Bioinformatics* 32: 292-294. <https://doi.org/10.1093/bioinformatics/btv566>

5. Li, Ruichao, Miaomiao Xie, Ning Dong, Dachuan Lin, Xuemei Yang, Marcus Ho Yin Wong, Edward Wai-Chi Chan, Sheng Chen. 2018. “Efficient generation of complete sequences of MDR-encoding plasmids by rapid assembly of MinION barcoding sequencing data.” *Gigascience* 7: 1-9. <https://doi.org/10.1093/gigascience/gix132>

6. Bankevich, Anton, Sergey Nurk, Dmitry Antipov, Alexey A Gurevich, Mikhail Dvorkin, Alexander S Kulikov, Valery M Lesin, et al. 2012. “SPAdes: a new genome assembly algorithm and its applications to single-cell sequencing.” *J Comput Biol* 19: 455-477. <https://doi.org/10.1089/cmb.2012.0021>

7. Koren, Sergey, Brian P Walenz, Konstantin Berlin, Jason R Miller, Nicholas H Bergman, Adam M Phillippy. 2017. “Canu: scalable and accurate long-read assembly via adaptive k-mer weighting and repeat separation.” *Genome Res* 27: 722-736. <https://doi.org/10.1101/gr.215087.116>

8. Ruan, Jue, Heng Li. 2020. “Fast and accurate long-read assembly with wtdbg2.” *Nat Methods* 17: 155-158. <https://doi.org/10.1038/s41592-019-0669-3>

9. Hu, Jiang, Zhuo Wang, Zongyi Sun, Benxia Hu, Adeola Oluwakemi Ayoola, Fan Liang, Jingjing Li, et al. 2024. “NextDenovo: an efficient error correction and accurate assembly tool for noisy long reads.” *Genome Biol* 25: 107. <https://doi.org/10.1186/s13059-024-03252-4>

10. Kolmogorov, Mikhail, Jeffrey Yuan, Yu Lin, Pavel A Pevzner. 2019. “Assembly of long, error-prone reads using repeat graphs.” *Nat Biotechnol* 37: 540-546. <https://doi.org/10.1038/s41587-019-0072-8>

11. Walker, Bruce J, Thomas Abeel, Terrance Shea, Margaret Priest, Amr Abouelliel, Sharadha Sakthikumar, Christina A Cuomo, et al. 2014. “Pilon: an integrated tool for comprehensive microbial variant detection and genome assembly improvement.” *PLoS One* 9: e112963. <https://doi.org/10.1371/journal.pone.0112963>

12. Hu, Jiang, Junpeng Fan, Zongyi Sun, Shanlin Liu. 2020. “NextPolish: a fast and efficient genome polishing tool for long-read assembly.” *Bioinformatics* 36: 2253-2255. <https://doi.org/10.1093/bioinformatics/btz891>

13. Wick, Ryan R, Louise M Judd, Claire L Gorrie, Kathryn E Holt. 2017. “Unicycler: Resolving bacterial genome assemblies from short and long sequencing reads.” *Plos Computational Biology* 13: e1005595. <https://doi.org/10.1371/journal.pcbi.1005595>

14. Shen, Wei, Shuai Le, Yan Li, Fuquan Hu. 2016. “SeqKit: A Cross-Platform and Ultrafast Toolkit for FASTA/Q File Manipulation.” *PLoS One* 11: e0163962. <https://doi.org/10.1371/journal.pone.0163962>

15. Wick, Ryan R, Mark B Schultz, Justin Zobel, Kathryn E Holt. 2015. “Bandage: interactive visualization of de novo genome assemblies.” *Bioinformatics* 31: 3350-3352. <https://doi.org/10.1093/bioinformatics/btv383>

16. Gurevich, Alexey, Vladislav Saveliev, Nikolay Vyahhi, Glenn Tesler. 2013. “QUAST: quality assessment tool for genome assemblies.” *Bioinformatics* 29: 1072-1075. <https://doi.org/10.1093/bioinformatics/btt086>

17. Marçais, Guillaume, Arthur L Delcher, Adam M Phillippy, Rachel Coston, Steven L Salzberg, Aleksey Zimin. 2018. “MUMmer4: A fast and versatile genome alignment system.” *Plos Computational Biology* 14:e1005944. https://doi.org/10.1371/journal.pcbi.1005944

18. Aziz, Ramy K, Daniela Bartels, Aaron A Best, Matthew DeJongh, Terrence Disz, Robert A Edwards, Kevin Formsma, et al. 2008. “The RAST server: Rapid annotations using subsystems technology.” *BMC Genomics* 9, 75. https://doi.org/10.1186/1471-2164-9-75

19. Bortolaia, Valeria, Rolf S Kaas, Etienne Ruppe, Marilyn C Roberts, Stefan Schwarz, Vincent Cattoir, Alain Philippon, et al. 2020. “ResFinder 4.0 for predictions of phenotypes from genotypes.” *J Antimicrob Chemother* 75: 3491-3500. <https://doi.org/10.1093/jac/dkaa345>

20. Carattoli, Alessandra, Ea Zankari, Aurora García-Fernández, Mette Voldby Larsen, Ole Lund, Laura Villa, Frank Møller Aarestrup, Henrik Hasman. 2014. “In silico detection and typing of plasmids using PlasmidFinder and plasmid multilocus sequence typing.” *Antimicrobial Agents and Chemotherapy* 58: 3895-3903. https://doi.org/10.1128/aac.02412-14

21. Alikhan, Nabil-Fareed, Nicola K Petty, Nouri L Ben Zakour, Scott A Beatson. 2011. “BLAST Ring Image Generator (BRIG): simple prokaryote genome comparisons.” *BMC Genomics* 12: 402. <https://doi.org/10.1186/1471-2164-12-402>

22. Sullivan, Mitchell J, Nicola K Petty, Scott A Beatson. 2011. “Easyfig: a genome comparison visualizer.” *Bioinformatics* 27: 1009-1010. <https://doi.org/10.1093/bioinformatics/btr039>
